# Antibiofilm and anticancer activities of unripe and ripe *Azadirachta indica* (neem) seed extracts

**DOI:** 10.1186/s12906-022-03513-4

**Published:** 2022-02-14

**Authors:** Kartik Chandra Guchhait, Tuhin Manna, Manas Barai, Monalisha Karmakar, Sourav Kumar Nandi, Debarati Jana, Aditi Dey, Suman Panda, Priyanka Raul, Anuttam Patra, Rittwika Bhattacharya, Subhrangsu Chatterjee, Amiya Kumar Panda, Chandradipa Ghosh

**Affiliations:** 1 Department of Human Physiology, Vidyasagar University, Midnapore, 721102 West Bengal India; 2 Department of Chemistry, Vidyasagar University, Midnapore, 721102 West Bengal India; 3 Department of Molecular Biology, Netaji Subhas Chandra Bose Cancer Research Institute, 3081 Nayabad, Kolkata, 700094 West Bengal India; 4 Department of Biophysics, Bose Institute, P-1/12 CIT Road, Scheme VIIM, Kankurgachi, Kolkata, 700054 West Bengal India; 5 Chemistry of Interfaces Group, Luleå University of Technology, SE- 97187 Luleå, Sweden; 6 Sadhu Ram Chand Murmu University of Jhargram, Jhargram, 721507 West Bengal India

**Keywords:** *Azadirachta indica*, Neem, Bacteria, *S. aureus*, *V. cholerae*, Biofilm, Cancer

## Abstract

**Background:**

Antibiotic resistances of pathogens and breast cancer warrant the search for new alternative strategies. Phytoextracts can eradicate microbe-borne diseases as well as cancer with lower side effects compared to conventional antibiotics.

**Aim:**

Unripe and ripe *Azadirachta indica* (neem) seed extracts were explored as potential antibiofilm and anticancer agents in combating multidrug-resistant infectious bacteria as well as anticancer agents against the MDR breast cancer cell lines.

**Methods:**

Shed-dried neem seeds (both unripe and ripe) were pulverized and extracted using methanol. The chemical components were identified with FTIR and gas chromatography - mass spectrometry. Antibiofilm activity of neem seed extracts were assessed in terms of minimum biofilm inhibitory concentration (MBIC), minimum biofilm eradication concentration (MBEC), and fluorescence microscopic studies on *Staphylococcus aureus* and *Vibrio cholerae*. Bacterial cells were studied by fluorescence microscopy using acridine orange/ethidium bromide as the staining agents. Minimum inhibitory concentration (MIC) and minimum bactericidal concentration (MBC) values were evaluated to observe the antibacterial activities. Cytotoxicity of the extracts against human blood lymphocytes and the anticancer activity against drug-resistant breast cancer cell lines were assessed by 3-(4,5-dimethylthiazol-2-yl)-2,5-diphenyltetrazolium bromide (MTT) assay and fluorescence-activated cell sorting (FACS) studies.

**Results:**

4-Ethyl-2-hydroxy-2-cyclopentene-1-one, phthalic acid, and 2-hexyl-tetrahydro thiophane were the major compounds in unripe neem seed, whereas 3,5-dihydroxy-6-methyl-2,3-dihydro-4-H-pyran-4-one and 4-ethylbenzamide were predominant in ripe neem seed. Triazine derivatives were also common for both the extracts. MBIC values of unripe and ripe neem seed extracts for *S. aureus* are 75 and 100 µg/mL, respectively, and for *V. cholerae*, they are 100 and 300 µg/mL, respectively. MBEC values of unripe and ripe seed extracts are 500 and 300 µg/mL, respectively for *S. aureus* and for *V. cholerae* the values are 700 and 500 µg/mL, respectively. Fluorescence microscopic studies at 16 and 24 h, after bacterial culture, demonstrate enhanced antibiofilm activity for the ripe seed extract than that of the unripe seeds for both the bacteria. MTT assay reveals lower cytotoxicity of both the extracts towards normal blood lymphocytes, and anticancer activity against breast cancer cell line (MDA-MB-231) with superior activity of ripe seed extract. FACS studies further supported higher anticancer activity for ripe seed extract.

**Conclusions:**

Methanolic extract of neem seeds could substantially inhibit and eradicate biofilm along with their potent antibacterial and anticancer activities. Both the extracts showed higher antibiofilm and antibacterial activity against *S. aureus* (gram-positive) than *V. cholerae* (gram-negative). Moreover, ripe seed extract showed higher antibiofilm and anticancer activity than unripe extracts.

**Graphical Abstract:**

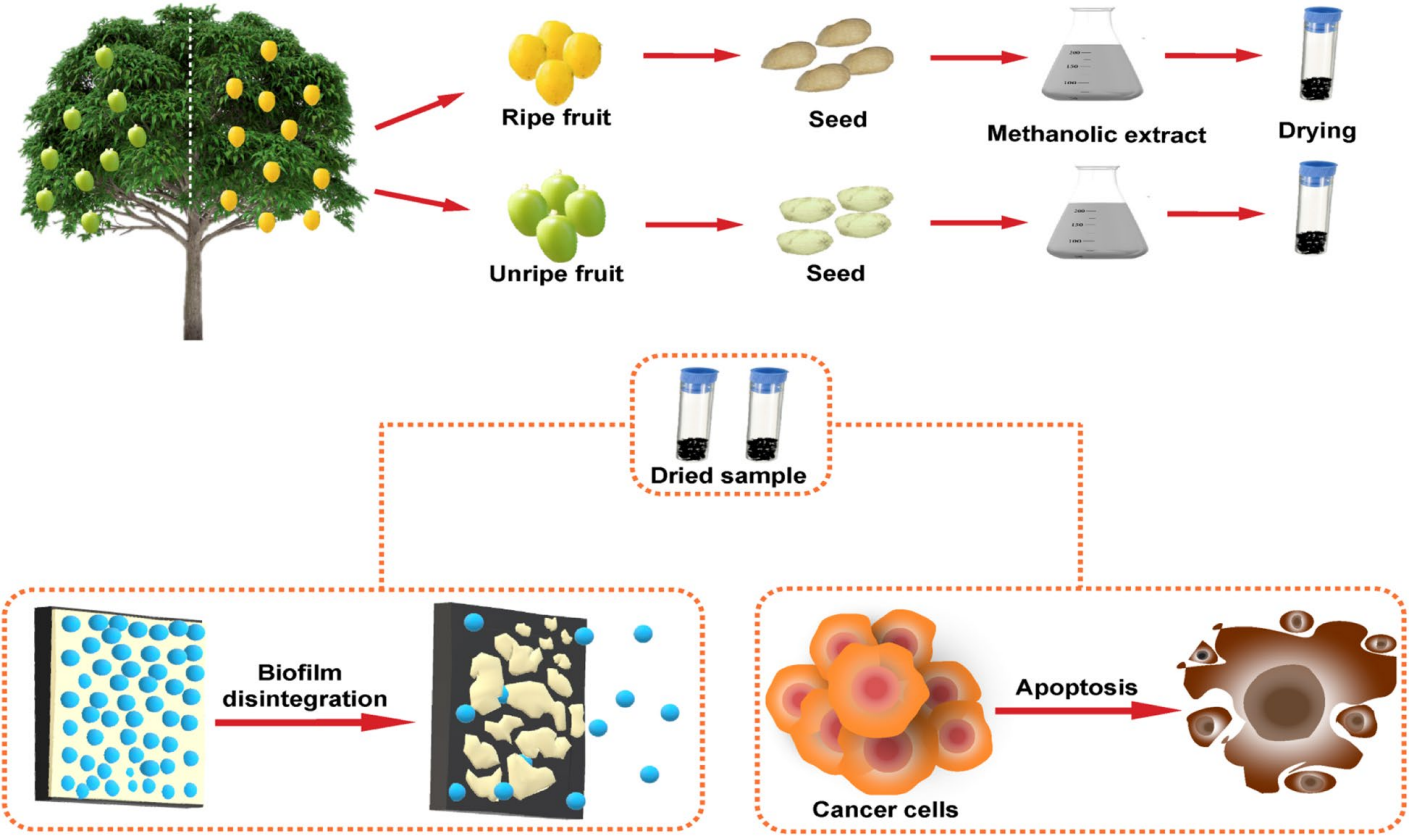

**Supplementary Information:**

The online version contains supplementary material available at 10.1186/s12906-022-03513-4.

## Background

Bacterial resistances to conventional antibiotics have emerged as one of the burning issues in therapeutic aspects. Remarkable escalation in the appearance of multidrug-resistant bacterial pathogens has caused a life-threatening emergent situation in the therapeutic arena and is responsible for the high rate of mortality, especially for immune-compromised individuals [1]. Development of host antibiotic resistance is caused mostly by their uncontrolled uses and this is mediated by increasing efflux pump activity, several modified enzymes which breaks the antibiotics and/or changing the target of antibiotics [2, 3]. Due to the enhanced resistance, it becomes harder to treat common infectious diseases [2, 4]. This situation is not only serious in medical indwelling-related hospital-acquired infections, but also been happening in serious magnitude in the communities [5, 6]. Therefore, effective development of alternative treatment strategies to antibiotics, chemotherapy, in various aspects like organ transplantation, surgeries, and cancer treatment is becoming a concern of towering demand to achieve control over the high risk of multi drug-resistant events. About 80% of bacterial infections are biofilm-mediated, the ability to form biofilm make the situation graver as bacterial cells in a biofilm can undermine host immune attack and restricts the antimicrobial substances to penetrate the biofilm layer [3, 6, 7].

Infections caused by *Staphylococcus aureus*, a gram-positive bacterial pathogen, have been indifferent to first-line antibiotics. *S. aureus* causes severe soft tissue infections in hospital settings and in communities [8, 9]. As per the report, methicillin-resistant *S. aureus* (MRSA) causes 64% more death globally than the non-resistant counterpart [8]. They cause acute infections like bacteraemia and skin abscesses by secreting several toxins and exo-enzymes [10]. In contrast, chronic infections are associated with a biofilm mode of growth where it can attach and persist on host tissues, such as bone and heart valves, to cause osteomyelitis and endocarditis, respectively, or on implanted medical indwellings, such as catheters, hip prosthetic implants, etc. [6, 10–12]. Among the food pathogens, *Vibrio cholerae* is a facultative anaerobic, gram-negative human pathogen. It causes pandemic/endemic cholera, cholera-like diarrhoea, and extra-intestinal infections [13, 14]. Consumption of contaminated water and/or food are the major causes of *V. cholerae* infections and reports reveal that around 1.3 to 4.0 million people get infected by them every year all over the world [15]. In inter-epidemic periods, *V. cholerae* can survive long in their natural habitats by virtue of their ability to form biofilm and hence, they could be potential threats in causing widespread infection in near future by evolving as an epidemic clone. Continued researches on emerging strains of *V. cholerae* have demonstrated a high frequency of emergence of multidrug-resistant strains [15, 16].

Report reveal that more than 60% infections caused by bacteria are biofilm based [17]. Biofilm is a cage-like structure formed by bacteria for its survival inside the host. This occurs through the creation of a matrix-like structure that covers and protects bacteria against different toxic agents or antimicrobial compounds as well as helps to access different nutrients [18, 19]. Consequently, biofilm formation in bacteria creates a suitable environment for their persistence and enhances their virulence properties [20, 21]. The extracellular matrix acts as a physical barrier that reduces the direct effects of antibiotics on bacterial cells [22, 23]. This physical barrier prevents antibiotics to enter into the core zone of biofilm. As a result, bacterium wins against the killer-antibiotics [24]. Common antibiotics normally kill planktonic cells and the majority of biofilm structures. However, the bacteria those can successfully survive even after the drug treatment, start a new cycle of biofilm development by repopulating the biofilm and disseminating into planktonic forms [25]. This eventually enhances the ailment process caused by biofilm-forming pathogenic microorganisms.

Beside the threat of antibacterial resistance, cancer is another health burden despite the improvement of diagnosis and modern surgical processes [26, 27]. Cancer cells are heterogeneous due to altered micro-environment and clonal evolution [28]. Under-developed and developing countries are more susceptible to cancer as a result of poor lifestyles and “westernized” diets [29]. It has been reported that 70% of cancer deaths occur in Africa, Asia, as well as in Central and South America [30]. Breast cancer is one of the most common cancers among women worldwide, accounting for approximately 5.7 million deaths [31]. Over 1.5 million women (25% of all women with cancer) are diagnosed with breast cancer every year globally [31, 32]. Breast cancer is metastatic and can commonly transfer to distant organs such as the bone, liver, lung, and brain, which mainly accounts for its incurability. Although chemotherapy and several anticancer drugs are available, their adverse side effects on normal cells/tissue, such as bone marrow function inhibition, nausea, vomiting, and alopecia lead scientists to search for some alternative therapies, which has little or no major health concern [33].

The aforementioned scenario has encouraged the present research group to make a target in identifying new bioactive compounds against the pathogenic bacterial infections as well as to target breast cancer. Inhibition of biofilm formation serves as a novel strategy to successfully combat bacteria bypassing the chance to generate bacterial resistance due to the minute or no pressure it exerts upon bacterial cell [34]. On the other hand, inhibition of proliferation and induction of cancerous cell death are the vital targeting area to control as well as to eradicate the cancerous cells [35]. Natural products have been playing important roles in exploring and processing of new drugs against bacterial infections and cancer development as their mode of action reduces the emergence of bacterial antibiotic resistance and abnormal cell progression, respectively [35, 36].

A growing number of evidences clarified that plant extracts possess considerable antimicrobial and anticancer potentials without any significant risk of resistance development against them [35, 37–39]. Besides, they are expected to have lower side effects. Plant-derived quinones, flavonoids, polyphenol, essential oils and tannins are known to complex with the cell wall synthesizing enzymes, inactivating it and thereby causes disruption of the microbial cell wall [40]. Alkaloids get intercalated into the membranes and destabilize them [40]. Polyphenols and tannins cause metal ion complexation and substrate deprivation to their target pathogens [41]. One of the rich sources of natural products is *Azadirachta indica* (neem) that has different beneficial properties in Indian as well as in African traditions [42]. Flowers, leaves, seeds, and bark of this plant have extensively been used as insecticide, antimicrobial, larvicidal, antimalarial, antibacterial, antiviral, and anticancer agent [43]. Components of neem are known to inhibit cancer progression as studied both *in-vitro* and *in-vivo* [44]. About 300 components have so far been identified from neem [45]. In spite of its tremendous potential, all the components and biological potentials are yet to be fully explored [45]. Proper comparative evaluation of unripe and ripe seed extracts is also so far illusive. Hence, the current study endeavours to delineate the potential of the methanolic extracts of neem seeds against two potent human pathogenic bacteria, i.e., *S. aureus* and *V. cholerae*, as antibiofilm agent, and also against breast cancer cells as anticancer agent. *S. aureus*, the Gram-positive and *V. cholerae*, the Gram-negative bacterial isolates were included in the study on the basis of their high biofilm forming abilities [46, 47]. A wide range of phytochemicals, both polar and nonpolar, can easily be extracted using methanol and also it has low boiling point that prevents the phytochemicals from damage during evaporation [48]. Antibiofilm activity of the extracts was assessed by spectrophotometric studies and fluorescence microscopy. MTT assessment was carried out on human blood lymphocytes to assess the biocompatibility of the extracts. Anticancer activity on MDA-MB-231 triple-negative breast cancer cell lines were carried out by combined methods of MTT and FACS analyses. Results from these analyses are expected to emphasize the future uses of neem seed against bacterial infection as the extract has lower side effects and easier availability.

### Experimental section

#### Materials

Methanol and potassium bromide (KBr) were purchased from Merck Limited, Mumbai, India. Roswell Park Memorial Institute medium (RPMI) and dimethyl sulfoxide (DMSO), 3-[4,5-dimethylthiazol-2-yl]-2,5-diphenyltetrazolium bromide (MTT), Luria-Bertani (LB) broth, and LB agar were procured from Hi-Media Laboratories, Mumbai, India. They were of AR grade and were used as received.

## Methods

### Collection of *Azadirachta indica* (neem) seed and extraction with methanol

Unripe and ripe *Azadirachta indica* fruits were collected in the month of April, 2019 from the campus of Vidyasagar University, Midnapore, West Bengal, India in two phases from the same tree. The plant was duly identified and authenticated by the Department of Botany, University of Calcutta, West Bengal, India vide accession no: CUH20098(CUH). All methods were performed in accordance with the relevant guidelines and regulations.

The unripe and ripe neem seeds were separately washed with water and then shed-dried for fifteen days at room temperature with little modification of method described previously [49]. 200 g dried seeds were then pulverized for 2 min using a blender. 50 g of powdered seed was mixed with 150 mL of HPLC grade methanol and incubated at 37ºC under shaking condition for 48 h. Methanol was evaporated from the filtrate of the extract. It was then dried under vacuum overnight to remove further traces of the solvent. The crystal-like crude extracts (2.2 and 2.6 gm of unripe and ripe extracts, respectively) were then stored in properly-labelled amber bottles at 4ºC under nitrogen atmosphere prior to use.

### FTIR studies

Identification of different functional groups in the methanolic extracts of both the unripe and ripe neem seeds were carried out by FTIR spectroscopy (Lambda, Perkin Elmer Spectrophotometer, USA) in the range of 3500- 400 cm^−1^ [50]. KBr disks containing solid extract material was prepared and used for FTIR studies.

### Gas Chromatography - Mass Spectrometry (GC-MS) studies

GC-MS analyses of neem seed extracts were performed on a Clarus 600 GC (Perkin Elmer, USA) System, fitted with a non-polar capillary column, coupled to a Clarus 600 C MS. Helium was used as the carrier gas at a constant flow rate of 1.0 mL/min [51]. The injection and ion source temperatures were 270 and 230ºC, respectively. The ionizing energy was set at 70 eV. Oven temperature was programmed from 60ºC (hold for 2 min) to 260ºC at a rate of 3 ºC/min. The crude samples were diluted with methanol (1:100, *v/v*) and filtered, particle-free dilute seed extracts were taken in a syringe and injected into the injector with a split ratio of 20:1. The mass spectra were computer matched with those of standards available in mass spectrum libraries (National Institute of Standards and Technology (NIST) Mass Spectral Library).

### Cytotoxicity assessment of phytoextracts against human blood lymphocytes

Blood samples were collected from healthy adult human volunteers by venipuncture in 5 mL heparin-containing tubes. Blood was collected from all human subjects with informed consent following all relevant guidelines and regulations. Experimental protocols in this regard were approved by Institutional Ethics Committee of Vidyasagar University, West Bengal, India. 5.0 mL of blood was diluted (1:1) with phosphate-buffered saline (PBS) and layered on Histopaque 1077 (Sigma-Aldrich, USA) by using a Pasteur pipette and centrifuged at 1500 rpm for 40 min at room temperature. The upper layer of the buffy coat (lymphocytes) was transferred using a clean Pasteur pipette to a clean centrifuge tube. It was washed three times and then resuspended in RPMI complete media supplemented with 10% fetal bovine serum (FBS) to incubate for a day at 37ºC in a 5% CO_2_ incubator [52]. Cytotoxicity of both unripe and ripe crude extracts were quantitatively estimated by colorimetric assay systems using MTT [53]. Optical density (OD) of the samples were measured on ELISA reader (BIO-RAD, Model 550, USA) using test and reference wavelengths of 570 and 630 nm, respectively. Percentage of cell viability was calculated by using the following equation [52]:

1 $$\mathrm{Cell}\;\mathrm{viability}\%=\left[{\mathrm{OD}}_{\mathrm{sample}}-{\mathrm{OD}}_{\mathrm{control}}\right]\times100/{\mathrm{OD}}_{\mathrm{control}}$$

### Bacterial strains and growth conditions

*Staphylococcus aureus* D1 and *Vibrio cholerae* TM8 bacterial strains were chosen from laboratory collections of present research group, based on their ability to produce high biofilm [46, 47, 54]. The *Staphylococcus aureus* D1 [46] and *Vibrio cholerae* TM8 [47] strains were isolated from pus and stool samples, respectively in clinical settings from hospitalized patients following research ethical guidelines and consent from appropriate authorities. For routine growth purposes, *S. aureus* and *V. cholerae* strains were cultured using Luria-Bertani (LB) broth and LB agar plates at 37ºC [54].

### Inhibition of biofilm formation

Effects of the methanolic extracts of unripe and ripe neem seeds on biofilm formation were tested by determining their minimum biofilm inhibitory concentration (MBIC) using the modified microdilution method [55]. Briefly, different concentrations of each methanolic neem seed extracts were prepared in such a way that the final concentration was in the range of 50 to 500 µg/mL.

Bacterial cultures were prepared by inoculating bacterial samples in test tubes containing 5mL LB broth and keeping it overnight in a shaking incubator at 37ºC. 5 µL of bacterial culture was added in 490 µL broth and 5µL of different concentrations of neem seed extracts (unripe and ripe, respectively) were separately added to test tubes so that the final volume of the tubes become 500 µL. PBS was used as negative control instead of the plant extracts. Samples were then incubated at 37ºC for 18 h in static condition. Modified crystal violet assay was performed to quantify biofilm formation [56]. OD values and the changes of biofilm ring in the LB tubes were shown in Table S1 and Fig. S2, respectively (Supplementary section). Biofilm inhibition percentage was calculated as follows [55]:

2 $$\mathrm{Inhibition}\%=\left({\mathrm{OD}}_{\mathrm{untreated}}-{\mathrm{OD}}_{\mathrm{treated}}\right)/{\mathrm{OD}}_{\mathrm{untreated}}\times100\%$$

### Inhibition of preformed biofilms

Biofilms were pre-formed in a borosilicate glass tube by aliquoting 50 µL of standardized *V. cholerae* and *S. aureus* (1.0 × 10^7^ CFU/mL) and incubated for 4 h at 37ºC. Sufficient biofilm biomass was observed within this time, as also supported by others (55). Following incubation, 50 µL of each of the neem seed extracts were added to the tubes. The tubes were further incubated for 24 h at 37 ºC [54]. After the incubation period, crystal violet assay was performed to quantify biofilm biomass [57]. OD values and the changes of biofilm ring in the LB tubes were shown in Table S2 and Fig. S3, respectively. Finally, minimum biofilm eradication concentration (MBEC) of the extracts was determined using Eq. (2).

### Antibacterial activities

MIC and MBC values were evaluated by the microdilution method in LB [58, 59]. The MBC values of the extracts were obtained by sub-culturing the MIC dilutions onto the sterile Mueller Hinton agar plates and incubated at 37ºC for 24 h. The lowest concentration of the extracts, in which no bacterial colony was found, and the bacteria were completely killed was considered as MBC.

### Fluorescence microscopic studies

Biofilms of both *V. cholerae* and *S. aureus* were visualized by fluorescence microscopy using 4,6-diamidino-2-phenylindol (DAPI), which stains the cells and extracellular matrix by passing through the cell membrane and allows the microscopic detection of the biofilm [60]. In brief, a glass surface was used as the substratum for biofilm development. Bacterial biofilms prepared on glass surface were washed with PBS once and covered with DAPI solution (0.01% *w/v*) for 10 min in the dark. The slide was then washed with PBS as well as sterile distilled water; and was dried before observation under a fluorescence microscope (Axio Scope A1, Zeiss, Oberkochen, Germany). To visualize the antibiofilm activity of both unripe and ripe extract, fluorescence imaging was performed using Axiocam 305 in the time gap of 8, 16, and 24 h for both the bacterial cells. To visualize and compare the antibiofilm activity of both extracts, the plant extracts were added in the same concentration (100 µg/mL) during the inoculation of each bacterium. Live and dead bacterial cells were examined by acridine orange (AO)/ethidium bromide (EB) staining with proper precautions for both *V. cholerae* and *S. aureus* to discriminate between the live (AO stained) and dead (EB stained) bacterial cells [61]. Overnight grown bacterial cells (107 cells/mL) were treated with unripe and ripe extracts (at their MIC values) for 15 min at 37ºC. After incubation, cells were harvested by centrifugation, washed with PBS, and stained with AO/EB (1:1) (100 mg/mL) for 30 min. After 30 min incubation, cells were washed with PBS and visualized under the fluorescence microscope using blue filter (480 nm).

### Cell culture

Human breast cancer cell line, MDA-MB-231 (ATCC® HTB-26™ RRID: CVCL_0062), extremely invasive breast carcinoma cell, was obtained from National Centre for Cell Science (NCCS), Pune, India. The cells were passaged at sub-confluence in T-25 flasks with Dulbecco’s modified eagle medium (DMEM) supplemented with 10% (*v*/*v*) heat-inactivated fetal bovine serum (Sigma-Aldrich), 2 mM glutamine, 1% (*v/v*) penicillin and streptomycin and maintained at 37ºC in a humidified incubator with 5% CO_2_. Low-passage cells were grown about approximately 70–80% confluency at previously described protocol [62, 63].

### Cell viability assay

Viable cell number was investigated through MTT assay [64]. The confluent MDA-MB-231 cells were plated in a concentration of 1 × 10^6^ cells/mL in a 96 well plate. After 24 h of growth, experimental cells were treated with variable concentrations of control drug (gemcitabine) and plant extracts (dissolved in DMSO) for 24 h. After drug-treatment, the cells were incubated with 10 µL of MTT (5 mg/mL) at 37 °C for 4 h. All the tests were performed in triplicate. Absorbance (at 570 nm) for each concentration was determined and their cell viabilities were calculated (in percentage, %) with respect to the blank [65, 66] using Eq. (1).

### Flow cytometric analysis of cell surface markers CD44 and CD326

Immunophenotyping is a useful method for the detection of cell surface-marker through flow cytometry or FACS. MDA-MB-231 cells (1.0 × 10^5^ cells/mL) were eroded with PBS and then harvested with trypsin-EDTA (Thermo Fisher Scientific, Ca # 25,300,054), at a concentration of 2.2 mM [67]. Ingathered cells were re-suspended in PBS with 0.5% fetal bovine serum with DMEM. To detect expression of fluorochrome-conjugated antibodieanti-CD44 (FITC-conjugated, Abcam, Ca # ab189524), and anti-CD326 (EpCAM, Abcam, Ca # ab239311) were added to the cell suspension, as recommended by the manufacturer, and incubated at 4 °C in the dark for 30–40 min. Cells without/with the drugs were washed and supernatants were aspirated out. Cells were then washed with sheath solution and incubated with antibody CD44, CD326 (EpCAM) in the dark for 15 min. Further, the cells were washed and dissolved in 500 μL sheath solution and finally analyzed using BD LSRFortessa^TM^ SORP (San Jose, CA) cell analyzer and BD FACS Diva v8.0.1 software as previously established protocol [67].

### Statistical analysis

The mean values and standard deviations of each quantitative variable were calculated after repeating each experiment three times. All the quantitative data were represented as mean ± standard deviation (SD). Statistical analyses were performed using Microsoft Excel. Two –tail Student’s t test was performed to compare the percent of biofilm inhibition/eradication by uniripe and ripe seed extracts for both bacteria. Differences were considered significant at *P*< 0.05.

## Results

### FTIR Analysis

**Fig. 1:**
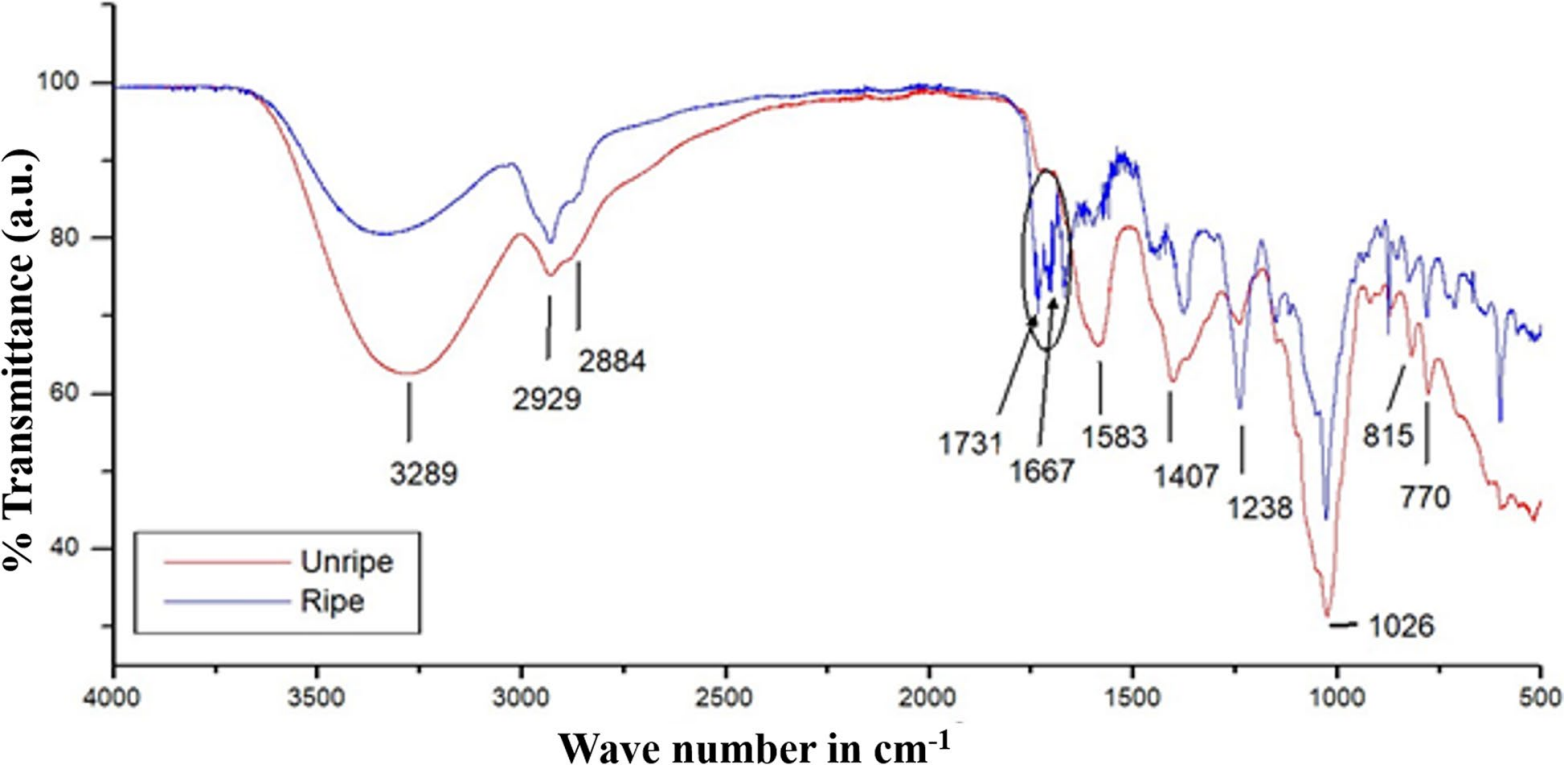
FTIR spectra of methanolic extract of unripe and ripe neem seeds

FTIR studies were performed to identify different functional groups in the methanolic extract of the seeds, as shown in Fig. 1. The C–O stretch in hydroxyl compound can be ascribed to the strong band that appeared at 1026 cm^−1^. The band around 1238 cm^−1^ is due to the presence of C=O groups in aromatic ester and C-N groups in amine moieties. In the unripe extract, the strong peak appeared at 1407 cm^−1^ that could be assigned to stretching vibrations of C-H and O-H bonds. The band around 1500-1600 cm^−1^ signifies aromatic rings C=C stretching vibration, enhanced by polar functional groups [68, 69]. Interestingly, only the ripe extract shows the characteristic band at 1667 cm^−1^ that represents the amide groups. Table 1 for ripe fruit reveals two amides (please see the following Sect. 3.2. GC-MS analyses), having peaks at 1662 and 1671 cm^−1^ (blue line in Fig. 1). The strong band appeared around 1731 cm^−1^ confirmed the presence of C=O bonds in ketones, aldehyde, and lactone [68]. Both the unripe and ripe seed extracts possess alkyl chain. The weak peak in both the samples had appeared at 2929 cm^−1^ for asymmetrical and at 2884 cm^−1^ for symmetrical stretching vibration can be attributed to C-H (methylene groups). The broad band around 3296 cm^−1^ was due to the O-H stretching mode of hydroxyl and N-H stretching mode of amine groups. Alkaloids, phenols, triterpenoids, tannins, oxalates, saponins, and flavonoids were previously reported in the methanolic neem seed extract [70].

**Table 1:** Chemical composition of methanolic extract of ripe neem seed detected by GC-MS analysis

Retention Time	Name of the Compound	% Area	MW (Da)	Molecular formula	Chemical class	Expected FTIR spectra (cm^−1^)	Biological activities
5.15	1-methylpyrrol	2.18	81	C_5_H_7_N	Heterocyclic compound	1509, 1266, 1090, 724, 606	Antimicrobial and anticancer activity [83]
17.12	3,5-dihydroxy-6-methyl-2,3-dihydro-4 H-pyran-4-one	11.41	144	C_6_H_8_O_4_	Flavonoids	1720, 1655	Antibacterial, antioxidant and anticancer activity [84]
26.38	2-hydroxy-n-3,3-trimethyl-butanamide	1.8	145	C_7_H_15_NO_2_	n-acyl amines	3366, 3164, 2962, 1662, 1634, 1430, 1418, 1263	
26.67	4-ethylbenzamide	4.75	149	C_9_H_11_NO	Phenolic compound	3343, 3168, 1671, 1618, 1414, 1398	Antitumor activity [85]

*All the compounds were selected according to the peak area percentage in the GC-MS data. Compounds were cross-checked with their expected FTIR spectra in Fig. 1

### GC-MS analyses

**Fig. 2:**
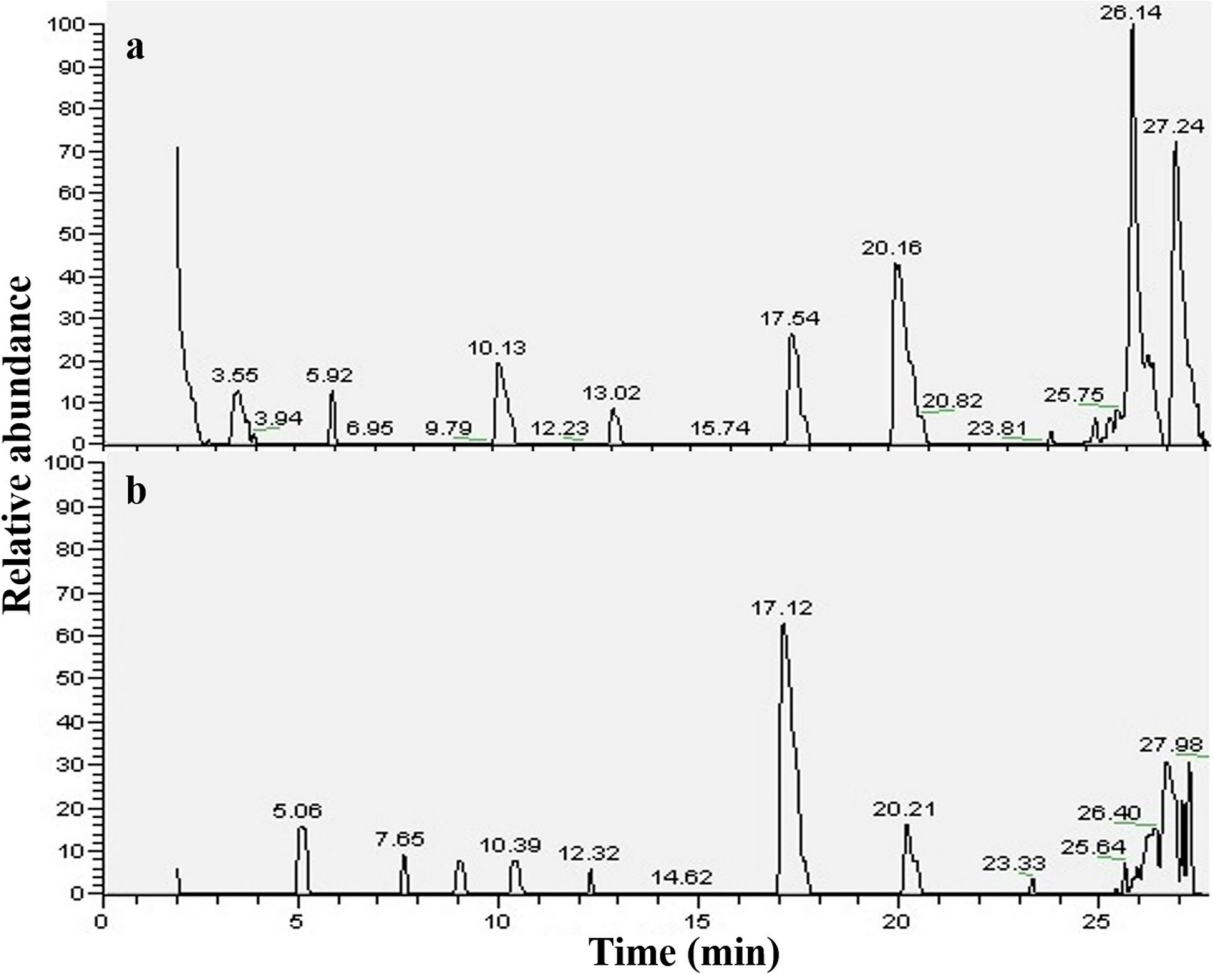
GC-MS chromatograms of methanolic extract of (**a**) unripe neem seeds, (**b**) ripe neem seeds

Methanolic extracts of both the unripe and ripe neem seeds were subjected to GC-MS analyses in identifying different active components (Fig. 2) and were further correlated with the FTIR data. In case of unripe neem seed, seven major compounds were detected as summarized in Table 2. 4-Ethyl-2-hydroxy-2-cyclopentene-1-one, phthalic acid, and 2-hexyl-tetrahydothiophane were the most abundant phytochemicals. Phthalic acid derivatives are reported to have antimicrobial activities both on gram-positive and gram-negative bacteria [71]. 2-Hexyl-tetrahydrothiophane has antimicrobial, anticancer as well as antifungal properties [72, 73]. On the other hand, four major compounds were found in the ripe seed extract (Table 1), in which 3,5-dihydroxy-6-methyl-2,3-dihydro-4 H-pyran-4-one and 4-ethylbenzamide were the predominant components. 3,5-Dihydroxy-6-methyl-2,3-dihydro-4 H-pyran-4-one is reported as potent antioxidant agent [74]. 4-ethylbenzamide has both antitumor and antimicrobial activities [75]. Triazine derivatives were found to be the common compound for both the extracts. The expected FTIR peaks of all the above-mentioned suggested compounds were listed in Tables 2 and 1. It was found that all these expected peaks are present in the recorded FTIR spectra (Fig. 1), a further support that the suggested compounds are indeed present in the extracts obtained.

**Table 2:** Chemical composition of methanolic extract of unripe neem seed detected by GC-MS analysis

Retention Time	Name of the Compound	% Area	MW (Da)	Molecular formula	Chemical class	Expected FTIR spectra (cm^−1^)	Biological activities
10.13	4-aminopyrimidine	3.48	95	C_4_H_5_N_3_	Pyrimidine alkaloids	3361, 3169, 1650, 1577, 1563, 1460, 804	Antibacterial activity [76]
13.02	2-oxo-2,3-dihydro-1 H imidazole-4-carbonitrile	1.05	109	C_4_H_3_N_3_0		3456-2778, 1642, 956	Antimicrobial activity and antioxidant activity [77]
17.53	1-napthyl acetoxy acetate	4.61	244	C_14_H_12_O_4_	Ester compound	1753, 1699, 1378, 1215, 1077, 1045, 777	Antibacterial activity [78]
20.16	4-ethyl-2-hydroxy-2-cyclopentene-1-one	8.65	126	C_7_H_10_O_2_	Carbonyl compound	2918, 1705, 1673, 1626, 1436, 1284, 1182, 840	Antibacterial activity [79]
26.14	phthalic acid	11.73	344	C_21_H_28_O_4_	Aromatic dicarboxylic acid	3094, 3013, 2896, 1701, 1687, 1678, 1405, 1283, 740	Antimicrobial activity [80]
26.55	3,7-dimethyl-(1,2,4)-triazolo-(4,3-b)-(1,2,4)-triazine	3.41	149	C_6_H_7_N_5_	Poly nitrogen containing heterocycles	1571, 1553, 1474, 1401	Anticancer activity [81]
27.24	2-hexyl-tetrahydrothiophane	13.55	172	C_10_H_2_0S	Heterocyclic compound	2963, 2951, 1454, 1440, 1254	Antimicrobial activity [82]

*All the compounds were selected according to the peak area percentage in the GC-MS data. Compounds were cross-checked with their expected FTIR spectra in Fig. 1

### Cytotoxicity studies

**Fig. 3:**
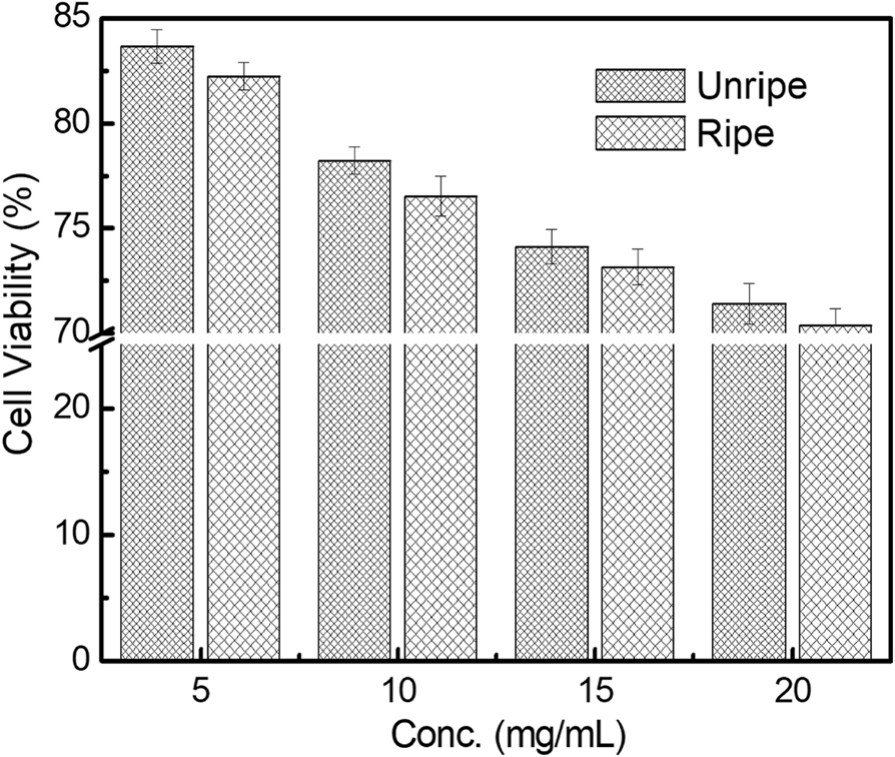
Cytotoxicity study of methanolic neem seed extracts on peripheral blood lymphocytes. Data are presented as mean ± SD

MTT assay on human blood lymphocytes was performed to determine the cellular toxicity levels of the methanolic extracts of neem seeds. At 5 mg/mL dosage, cell viabilities were 83.65 and 82.22% for unripe and ripe extracts, respectively. At the highest dosage (20 mg/mL), the cell viabilities were 71.42 and 70.38%, respectively for unripe and ripe neem extracts (Fig. 3). No significant morphological difference of the lymphocytes was seen under light microscope (Fig. S1, Supplementary section). To use the phytoextracts as antibiofilm agents, it preferably should have low cytotoxicity. Results clearly indicate that the extracts can safely be used for future studies at substantially higher concentrations.

### Minimum biofilm inhibitory concentration (MBIC)

**Fig. 4:**
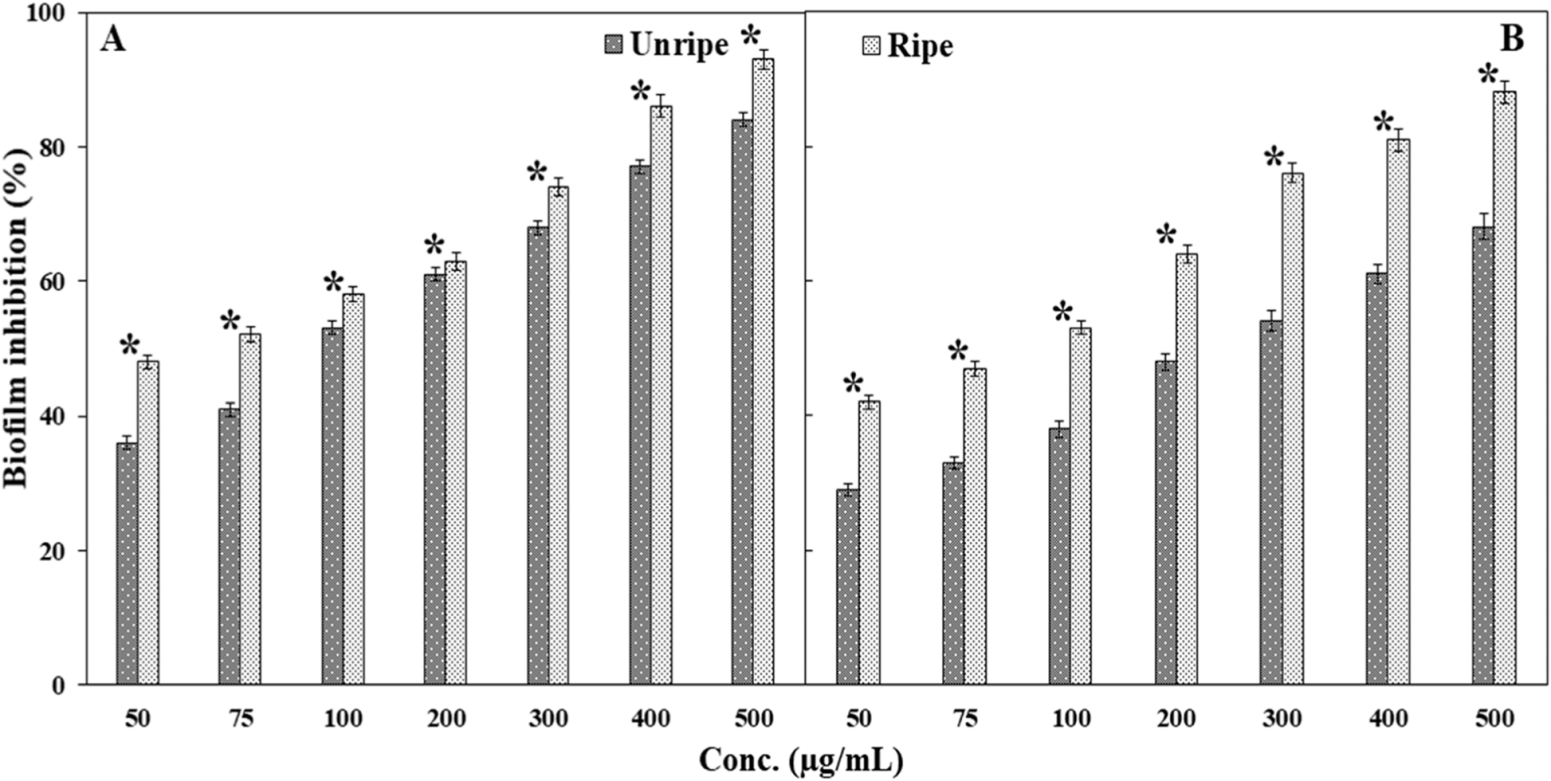
Inhibitory effects of methanolic neem seed extracts on biofilm formation in (**A**) *S. aureus*, and (**B**) *V. cholerae*, expressed as percentage inhibition. Reduction of biofilm formation ≥ 50% is considered as the MBIC value. Data are presented as mean ± SD. Two-tail Student’s t test was performed to compare the percent of biofilm inhibition caused by uniripe and ripe seed extracts for both bacteria. * denotes significant difference at *P*< 0.05

The minimum concentration that inhibits the formation of biofilm by ≥ 50% is considered as the MBIC [86]. MBIC values of the unripe and ripe neem seed extracts on *S. aureus* were 100 and 75 µg/mL, respectively (Fig. 4 A). In the case of *V. cholera*e, it was 300 and 100 µg/mL, respectively (Fig. 4B). MBIC of vancomycin against *S. aureus* has been reported to be 16 µg/mL [87]. Results suggest that the ripe seed extract was more potent as an antibiofilm agent as they could show significant higher percentage of inhibition of biofilm (*P*<0.05) at all the concentrations than unripe seed extract. Though both kinds of seed extracts exhibited inhibitory effects on biofilm formation on both the bacteria, antibiofilm efficacy was found to be higher in case of *S. aureus* than *V. cholerae* (Fig. 4). It, therefore, could be concluded that ripening may have transformed certain bioactive components, and those might be responsible for such outcome. However, further thorough studies are required in identifying the components through other analytical tools.

### Minimum biofilm eradication concentration (MBEC) studies

**Fig. 5:**
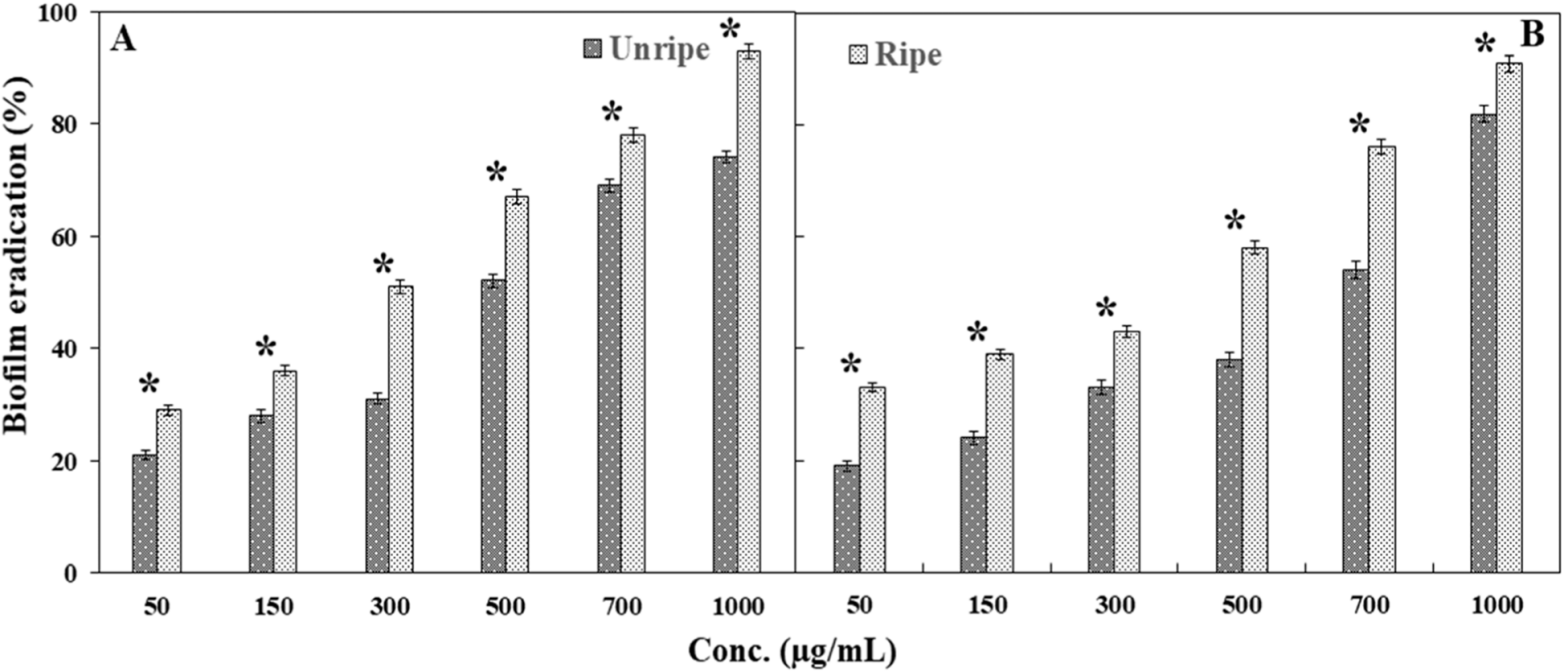
Biofilm eradication activity of methanolic neem seed extracts on preformed biofilm of (**A**) *S. aureus*, (**B**) *V. cholerae*, expressed as percentage inhibition. Eradication ability of ≥ 50% of preformed biofilm is the MBEC dosage. Data are presented as mean ± SD. Two –tail Student’s t test was performed to compare the percent of biofilm eradication caused by uniripe and ripe seed extracts for both bacteria. * denotes significant difference at *P*< 0.05

MBEC is defined as the concentration that shows ≥ 50% eradication of preformed biofilm [88]. MBEC values of methanolic unripe neem seed extract were 500 and 700 µg/mL for *S. aureus* (Fig. 5 A) and *V. cholerae* (Fig. 5B), respectively. On the other hand, in the case of ripe neem seed extract, MBEC were 300 and 500 µg/mL for *S. aureus* and *V. cholerae*, respectively (Fig. 5). Both unripe and ripe extracts showed higher biofilm eradication efficiency for *S. aureus* than *V. cholerae.* Here also results suggest that the ripe seed extract is more effective as biofilm eradication agent as they could show significant higher percentage of eradication of preformed biofilm (*P*<0.05) at all the concentrations than unripe seed extract. MBEC of vancomycin against *S. aureus* is > 256 µg/mL [87] and that for doxycycline is 32 µg/mL against *V. cholerae* [89].

### Antibacterial activity

Apart from exploring the antibiofilm activity, the ability of neem seed extracts to inhibit bacterial growths was also studied. MIC value is the lowest concentration that lacks turbidity and is fully transparent and corresponds to 99% of bacterial growth inhibition [90]. MIC values of ripe neem seed extracts are 1.2 and 2.0 mg/mL and MBC values are 2.4 and 4.0 mg/mL for *S. aureus* and *V*. *cholerae*, respectively. MIC values of unripe neem seed extracts are 6.0 and 8.5 mg/mL, and MBC values are 12.0 and 17.0 mg/mL for *S. aureus* and *V. cholerae*, respectively. Results indicate that ripe neem seed extracts possess higher antibacterial activity than the unripe seed extracts against the specified bacterial strains. Ripening of the neem seed altered the secondary metabolite synthesis and from the GC-MS study, it has been observed that some phytochemicals, viz., 5-dihydroxy-6-methyl-2,3-dihydro-4 H-pyran-4-one and 4-ethylbenzamide is present in high percentage in the ripe seed than unripe seed extract, supposed to be responsible for such effect. MIC and MBC values of vancomycin against *S. aureus* are 1.0 and 2.0 µg/mL, respectively [87]. Whereas, MIC and MBC values of doxycycline against *V. cholerae* are 0.25 and 2 µg/mL respectively [89].

### Visualization of biofilm with fluorescence microscope

**Fig. 6:**
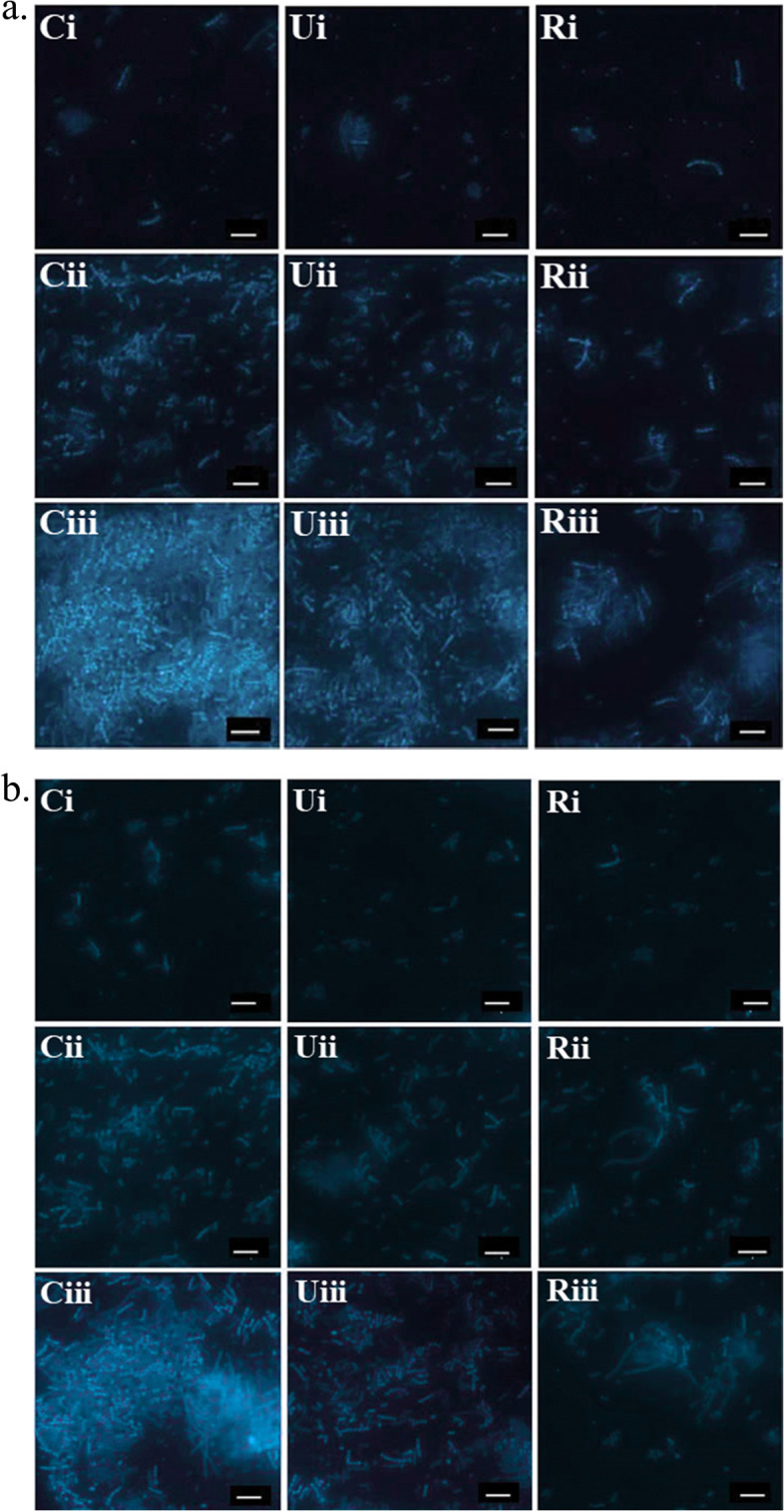
Fluorescence microscopic images of DAPI stained (**a**) *S. aureus*, and (**b**) *V. cholerae*, after treatment with neem seed extracts. Vertical panels Ci, Cii and Ciii correspond to biofilm biomass at 37˚C for 8, 16 and 24 h, respectively; Vertical panel Ui, Uii and Uiii correspond to biofilm biomass treated with unripe neem seed extract for 8, 16 and 24 h, respectively; and, vertical panel, Ri, Rii and Riii correspond to biofilm biomass treated with ripe neem seed extract for 8, 16 and 24 h, respectively. Scale bar: 10 μm

Biofilm formed by the bacterial strains were documented with fluorescence microscopic studies after staining with DAPI [60]. Fluorescence intensities were measured as relative fluorescent unit (RFU) using online ImageJ software (ImageJ, NIH, Bethesda, Maryland, USA) and fold changes of the intensity against corresponding control system have been shown in Table S3. The control group without neem seed extract for both the bacteria produce high biofilm, whereas bacterial cells (both for *S. aureus* and *V. cholerae*) treated with unripe and ripe neem seed extracts showed moderate and low biofilm, respectively (Fig. 6). In the case of high biofilm, green fluorescent light emission was intense and for treated groups (with unripe and ripe neem seed extracts), the intensity of light emission was moderate to less.

**Fig. 7:**
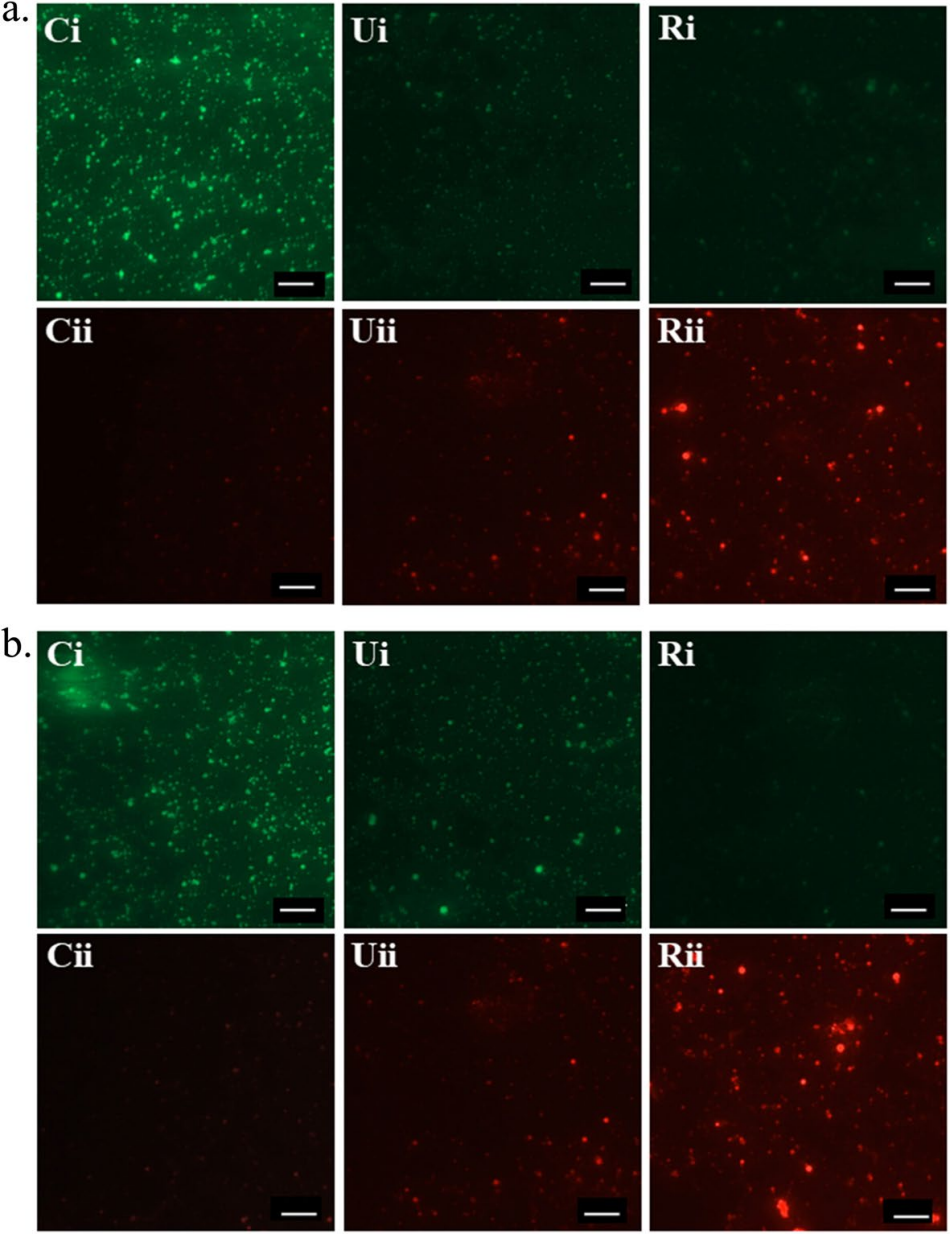
Fluorescence microscopic images of bacterial cell death via AO/EB staining of (**a**) *S. aureus*, and (**b**) *V. cholerae*, after treatment with neem seed extracts. Each longitudinal panel in both figures indicates AO (Acridine Orange) and EB (Ethidium Bromide) stained live and dead bacterial cells, respectively. Live bacteria emit green fluorescence and dead bacteria emit red fluorescence. Vertical panel Ci and Cii corresponds to bacterial growth at 37˚C for 18 h after AO and EB staining, respectively; Vertical panel Ui and Uii correspond to bacterial growth treated with unripe neem seed extract after AO and EB staining, respectively; Vertical panel Ri and Rii correspond to bacterial growth treated with ripe neem seed extract after AO and EB staining, respectively. Scale bar: 20 μm

Live and dead bacterial cells were visualized by AO/EB staining. In case of AO/EB staining, AO stains the live cells while the dead bacterial cells which have lost membrane integrity get stained by EB. Representative fluorescence micrographs are shown in Fig. 7 and live/dead bacterial cell number was counted (using ImageJ software) and fold changes against corresponding control system have been shown in Table S4. For comparative analysis of antibacterial activity, lower MIC dosage between the unripe and ripe extract (1.2 mg/mL for *S. aureus* and 2.0 mg/mL for *V. cholerae*) were selected. The untreated cells result in maximum green fluorescence for both *S. aureus* and *V. cholerae*, where green fluorescence corresponds to live bacterial cells. Bacterial cells treated with unripe neem seed extracts exhibit moderate red fluorescence, whereas bacteria treated with ripe neem seed extracts show significantly high red fluorescence (Fig. 7). Dead bacterial cells correspond to red fluorescence as EB can cross the cell membrane of those dead bacteria.

### Anticancer activity

**Fig. 8:**
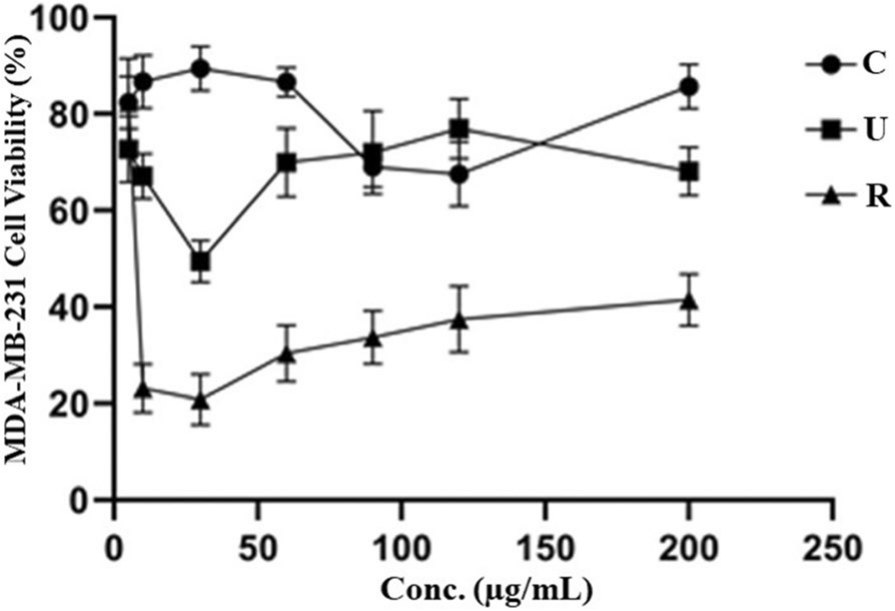
Evaluation of cell viability of multidrug-resistant breast cancer cell MDA-MB-231 treated with control drug gemcitabine (C), unripe neem seed (U) and ripe neem seed (R) extract by MTT assay. IC_50_ dosage against selected cancer cells for control drug Gemcitabine (C) was found to be 200 µg/mL, whereas the same for unripe (U) and ripe (R) seed extracts were 30 µg/mL and 10 µg/mL, respectively

Individually, extracts could significantly diminish the viability of MDA-MB-231 breast cancer cells for 24 h treatment condition and ensured 50% inhibition concentration (IC_50_) of cell proliferation. The IC_50_ of cell proliferation is dose-dependent. The IC_50_ effect of plant extract on the cell growth of MDA-MB-231 is illustrated in Fig. 8. Results revealed that unripe neem seed extract and ripe neem seed extract reflected 50% inhibitory effect at IC_50_ of 30 µg/mL (SD ± 2.3) and 10 µg/mL (SD ± 1.6), respectively (Fig. 8). The IC_50_ of gemcitabine (standard drug) could not show 50% inhibition in concentration of cell proliferation. In Fig. 8, gemcitabine could almost 28% expressively decrease the viability of MDA-MB-231 in similar treatment condition and the concentration was found to be 125 µg /mL. Similar observations were also obtained by Moongkarndi et al., 2004, where the proliferation of SKBR3 breast cancer cells were inhibited by 50% with 9.25 µg/mL crude methanolic extract of *Garcinia mangostana* [91]. Fig. S4 indicated reduced number of MDA-MB-231 breast cancer cells as well as slight morphological alteration in the ripe neem seed extract treated group during the MTT assay. Although, no significant observable difference in cancer cell numbers was seen in between the unripe neem seed treated and gemcitabine treated groups.

### Immunophenotyping analysis by FACS studies

**Fig. 9:**
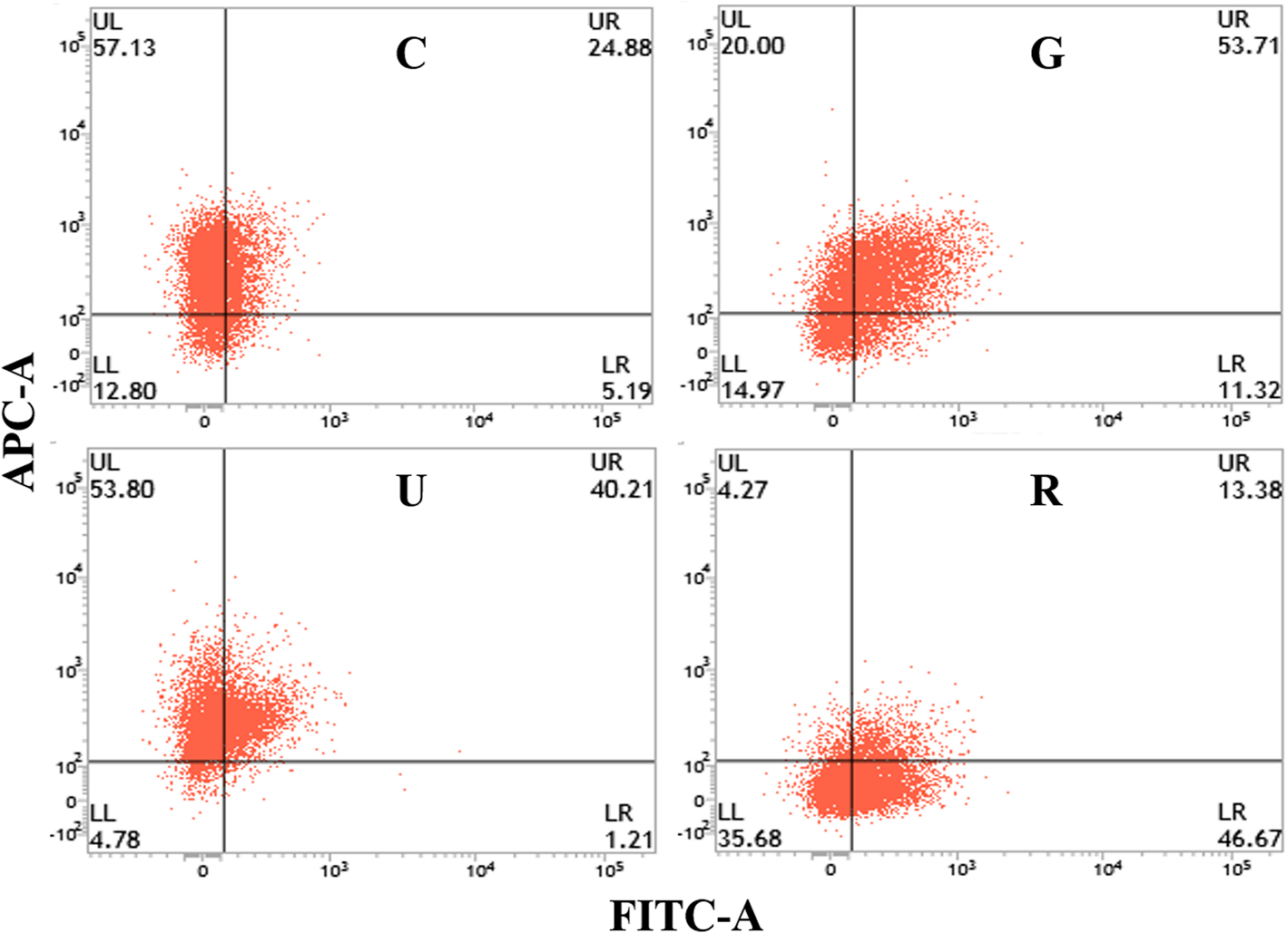
The fate of MDA-MB-231 CD44 positive (FITC conjugated) and EpCAM positive (APC conjugated) breast cancer stem cell line as, C: cell line without any treatment, G: cell line treated with Gemcitabine, U: cell line treated with unripe neem seed extract, and R: cell line treated with ripe neem seed extract

Expression of the indispensable markers for breast cancer cell lines, CD44 and CD326 in MDA-MB-231 cells without/with the plant extract treatment were also studied through flow cytometry analysis. The cells were also treated with gemcitabine, a regular chemotherapeutic drug, as the positive control drug in similar treatment conditions where the drug was not found to function significantly in respect of untreated cells. As shown in Fig. 9, after 24 h of treatment with 10 µg/mL ripe seed extract, the level of both the marker populations decreased with respect to the untreated and positive control. In the case of unripe seed extract, both the markers were decreased from the control.

## Discussions

Growing evidences of drug resistance in bacteria and several side effects of the conventional chemotherapeutic processes of cancer treatment have motivated the researchers to explore some alternative but convenient approaches for dealing of infections and cancer [92, 93]. It is known that in bacterial pathogenesis, the ability of bacterial cells to cause infection gets potentiated by conforming biofilms, based on quorum sensing (QS) signaling action. Hence, the inhibition of biofilm-forming ability as well as QS, the cell-cell communication becomes a promising alternative as a controlling strategy for these healthcare issues [8, 94]. Under such circumstances, the identification and establishment of alternative agents to antibiotics are warranted to combat bacterial infections [95]. With that intention, the present study was designed to explore the effect of methanolic extract of unripe and ripe neem seeds on biofilm formation and eradication. Exploring anticancer activity of the neem seed extracts on MDR breast cancer cells was also another significant part of this study. Thiophene derivative compounds are known to have potential remedial properties to certain biofilm-related bacterial infections [96]. In the present study, GC-MS analyses revealed the presence of 2-hexyl-tetrahydrothiophane in unripe methanolic neem seed extract, considered to be responsible for antibiofilm activity towards the target bacteria. Ripe neem seed extracts containing highest percentage of 3,5-dihydroxy-6-methyl-2,3-dihydro-4 H-pyran-4-one and 4-ethylbenzamide showed significantly higher (*P*<0.05) antibiofilm activity, as revealed through the MBIC, MBEC and fluorescence studies, than the unripe neem seed extract. 3,5-dihydroxy-6-methyl-2,3-dihydro-4 H-pyran-4-one has been reported to have potent antioxidant and antimicrobial activities [97, 98]. On the other hand, the antibacterial activity of 4-ethylbenzamide has already been described [99]. It was also found that antibiofilm activities of both the extracts were higher in the case of *S. aureus* than *V. cholerae*. The differential structural properties as well as biofilm regulatory properties in gram-positive and gram-negative bacteria might be the underlying causes for such observations. Besides, it may be suggested that plant extracts may play some roles in modulating cell wall synthesizing enzymes and also QS cell-cell communication and regulation during biofilm formation to induce antibiofilm activity. Moreover, 3,5-Dihydroxy-6-methyl-2,3-dihydro-4 H-pyran-4-one, a derivative of kaempferol, is present in highest percentage in ripe neem seed extract. Kaempferol is clinically known to possess anticancer, antimicrobial and antioxidant activities [100, 101]. Kaempferol derivatives and its synergistic action along with other compounds present in the seed extracts, is considered to be responsible for possessing significantly higher antibiofilm as well as anticancer activity in methanolic ripe neem seed extracts than unripe neem seed extract. MIC and MBC results also indicate that ripe neem seed extracts possess greater antibacterial activity than unripe extracts against both tested gram-positive (*S. aureus*) and gram-negative (*V. cholerae*) bacteria. In this context it may also be considered that some antibiotics exhibit antibiofilm activities, but report reveals that conventional antibiotics are not prudent enough against bacterial biofilm [102]. Also, combination strategies, involving different antimicrobial peptides (AMPs) are being used along with different conventional antibiotics [102]. Besides, numerous phytochemicals are being used as antimicrobial and antibiofilm agents. Hence depending on several reports, it appears that effective therapeutic outcome along with lesser side effects and lower propensity for resistance development of the phytochemicals validate their usefulness as an alternative to antibiotics and other chemotherapeutics [103, 104].

MTT analysis showed the reduced viability of the breast cancer cell line, whereas, FACS study emphasizes that both the CD44 and CD326 populations of the breast cancer cells have been significantly decreased. CD44 and CD326 are cell surface markers. The population of these markers significantly increased during the cancer prognosis and, induces cell signalling, proliferation, differentiation and migration of the cancer cells [105, 106]. Here, Ripe seed extract possessed significantly greater killing potency against the cancer cell line than unripe extract. Several studies on 3,5-dihydroxy-6-methyl-2,3-dihydro-4 H-pyran-4-one showed its antimicrobial and anti-proliferative activity [98], and 4-ethylbenzamide, derivatives of 4-thiazolidinone conferred their anticancer potency [107]. It was also observed that ripe seed extract has superior anticancer activity than that found with gemcitabine and unripe seed extract. Unripe seed extract could reduce the population of cancer cells similar to the magnitude of gemcitabine-based inhibition. It may be suggested from the present study that neem seed extracts have some role in the inhibition of cancer cell proliferation and differentiation as well as induction of apoptosis process in cancer cells. Further studies are warranted for identification and characterization of the particular phytochemical responsible for the antibiofilm and anticancer activities. It is also necessary to pinpoint the component transformed during ripening of the seeds, considered to be responsible for the enhanced antibiofilm and anticancer activities.

Though the effective concentrations of the extracts were relatively higher than the conventional antibiotics, it should also be considered that these extracts contain several components and the effect of each of the components are needed to be investigated. The next approach will be to identify the individual components responsible for such effects along with tracking the exact pathway by which they exert their action. When all these factors are taken into account, it will certainly be helpful in the development of some new potent alternatives to the conventional antibiotics. Besides, easy availability, low cost, fewer side effects and long history of use in folk medicine for curing several infections and disease, are considered to make plant extracts as good resource for investigations.

## Conclusions

Antibiofilm studies of neem seed extracts against *S. aureus* and *V. cholerae* show that the methanolic extracts of neem seeds are potentially effective against the formation and eradication of bacterial biofilm. Above a certain concentration, both the unripe and ripe seed extracts are capable to cease bacterial growth. Interestingly, these phytoextracts also possess potential anticancer activity on MDA-MB-231 breast cancer cell line. Methanolic extracts of ripe neem seed have significantly greater efficiency than the unripe neem seed. It could be due to the secondary metabolites of neem seed during the process of ripening. These key findings indicate that both unripe and ripe seed extracts can be beneficial for anti-virulence strategies through anti-biofilm potential against numerous pathogenic bacteria. Simultaneously the ability of these extracts to destroy cancer cells opens a new dimension in cancer therapeutics. The present results clearly indicate that the methanolic extracts of both unripe and ripe neem seeds can safely be used for acting as antibiofilm and anticancer agents in modern phytomedicine. However, ripe seed extract showed higher antibiofilm, antibacterial and anticancer activities. Furthermore, identification and exploration of the active compounds behind such activities are needed.

## Supplementary Information

**Additional file 1.**

## Data Availability

All data generated or analyzed during this study are included in this article and its supplementary information files. Additionally, this study does not have any mandate data.

## Abbreviations

MRSA: Methicillin-resistant *staphylococcus aureus*
RPMI: Roswell park memorial institute medium
PBS: Phosphate-buffered saline
DMSO: Dimethyl sulfoxide
MTT: 3-(4,5-Dimethylthiazol-2-yl)-2,5-diphenyl tetrazolium bromide
FTIR: Fourier transform infrared spectroscopy
MBIC: Minimum biofilm inhibitory concentration
MBEC: Minimum biofilm eradication concentration
MIC: Minimum inhibitory concentration
MBC: Minimum bactericidal concentration
DAPI: 4,6- Diamidino-2-phenylindole
DMEM: Dulbecco’s modified eagle medium
FACS: Fluorescence activated cell sorting
CLSI: Clinical & laboratory standards institute
