# Supplementary material for: Antibiofilm and anticancer activities of unripe and ripe Azadirachta indica (neem) seed extracts
Source: BMC Complement Med Ther. 2022 Feb 14;22:42. doi: 10.1186/s12906-022-03513-4 (PMC8843028; doi:10.1186/s12906-022-03513-4)
Supplement: Supplementary file 1 — Additional file 1. [file 12906_2022_3513_MOESM1_ESM.pdf]

Antibiofilm and Anticancer Activities of Unripe and Ripe *Azadirachta indica* (neem) Seed Extracts

Submission ID -bba31bf8-4a90-4c1a-81fb-35a8e398c33e

Supplementary Section:

Table S1. OD values at 570 nm wavelength for the determination of MBIC of unripe and ripe neem seed extracts against *S. aureus* and *V. cholerae*. $OD_{control}$ for *S.aureus* was 1.241 and, that for *V.cholerae* was 1.132.

**Doses (µg/mL)**	**OD_{570}**			
	**Unripe neem seed extract**		**Ripe neem seed extract**	
	S. aureus	***V. cholera***	***S. aureus***	***V. cholere***
50	0.795	0.803	0.645	0.656
75	0.732	0.758	0.595	0.598
100	0.583	0.701	0.521	0.532
200	0.482	0.589	0.459	0.407
300	0.397	0.521	0.323	0.271
400	0.285	0.441	0.173	0.215
500	0.198	0.362	0.087	0.136

Table S2. OD values at 570 nm wavelength for the determination of MBEC of unripe and ripe neem seed extracts against *S. aureus* and *V. cholerae*. $OD_{control}$ for *S.aureus* was 1.214 and, that for *V.cholerae* was 1.125.

**Doses (µg/mL)**	**OD_{570}**			
	**Unripe neem seed extract**		**Ripe neem seed extract**	
	S. aureus	***V. cholera***	***S. aureus***	***V. cholere***
50	0.966	0.912	0.869	0.753
150	0.882	0.855	0.783	0.687
300	0.844	0.753	0.598	0.641
500	0.587	0.697	0.403	0.472

700	0.379	0.517	0.269	0.271
1000	0.318	0.203	0.085	0.101

Table S3. Fluorescence intensities of Ui, Ri, Uii, Rii, Uiii and Riii in respect to their control system from Fig. 6, were measured as Relative Fluorescent Unit (RFU) using imageJ software, and fold changes from their respective control system have been tabulated.

	**Bacteria**	**Fluorecence intensity change from their corresponding control (fold change)**
Ci	*S. aureus*	
	V. cholerae	
Ui	*S. aureus*	1.08
	V. cholerae	1.03
Ri	*S. aureus*	1.16
	V. cholerae	1.07
Cii	*S. aureus*	
	V. cholerae	
Uii	*S. aureus*	1.35
	V. cholerae	1.08
Rii	*S. aureus*	1.47
	V. cholerae	1.31
Ciii	*S. aureus*	
	V. cholerae	
Uiii	*S. aureus*	2.06
	V. cholerae	1.59
Riii	*S. aureus*	3.14
	V. cholerae	2.56

Table S4. Fold change of live/dead bacterial numbers comparing with their respective control system from Fig. 7, have been tabulated. ImagJ software was used to measure this fold changes.

		**Live bacterial cell number**	**Reduction of live bacterial cell number (fold change)**		**Dead bacterial cell number**	**Increment of dead bacterial cell number (fold change)**
S. aureus	Ci	953		Cii	98	
	Ui	582	1.63	Uii	468	4.97
	Ri	130	7.33	Rii	822	8.38
V. cholerae	Ci	788		Cii	144	
	Ui	502	1.56	Uii	388	2.69
	Ri	170	4.63	Rii	898	6.23

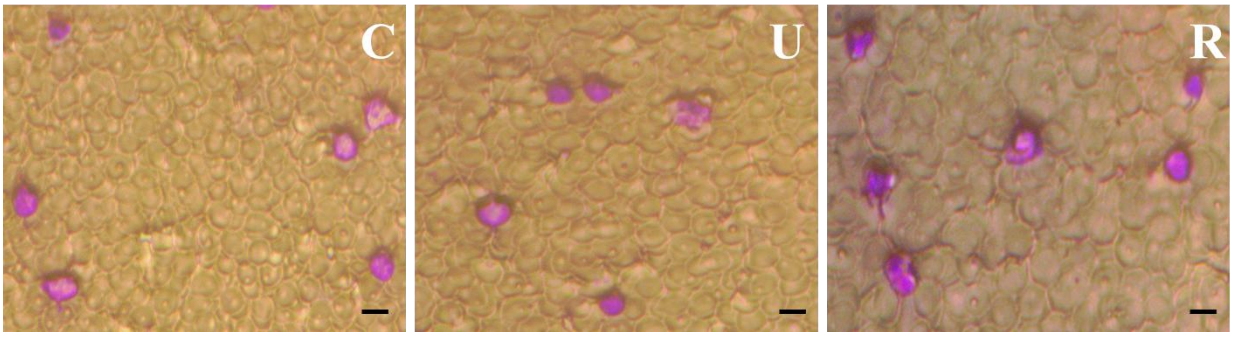

Fig. S1. Leishman-stained films of human blood lymphocytes in the cytotoxicity study. Lymphocytes showing no detectable morphological change after the treatment with unripe (U) and ripe (R) neem seed extracts at their highest dose (20 mg) applied. Lymphocytes treated without any seed extracts were used as positive control (C). Scale bar: 10 µm.

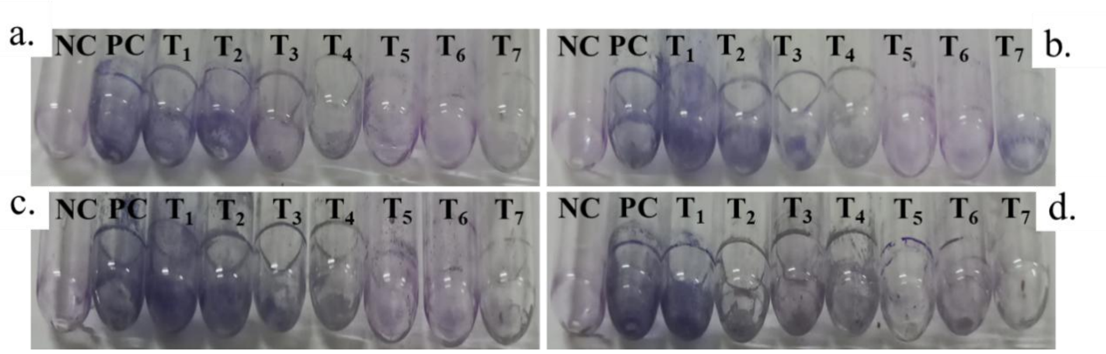

Fig. S2. Crystal violet biofilm assay in borosilicate tubes for the estimation of MBIC of unripe (a & b) and ripe (c & d) neem seed extracts against *S. aureus* (a & c) and *V. cholerae* (b & d). NC: Negative control containing only LB broth; PC: Positive control containing LB broth with the respective bacteria; T_1-T_7: LB broth containing different concentrations of unripe and ripe neem seed extracts, respectively for each bacteria used in this study.

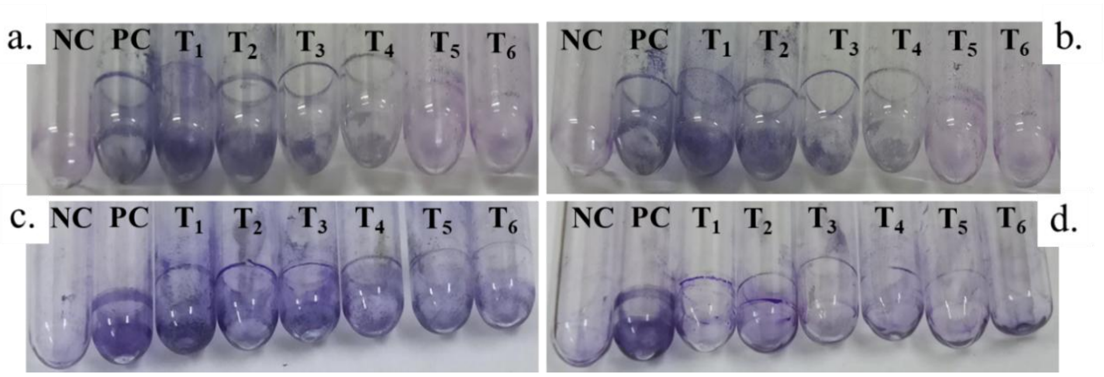

Fig. S3. Crystal violet biofilm assay in borosilicate tubes for the estimation of MBEC of unripe (a & b) and ripe (c & d) neem seed extracts against *S. aureus* (a & c) and *V. cholerae* (b & d). NC: Negative control containing only LB broth; PC: Positive control containing LB

broth with the respective bacteria; T_1-T_7: Bacterial preformed biofilm treated with different concentrations of unripe and ripe neem seed extracts, respectively for each bacteria used in this study for biofilm eradication.

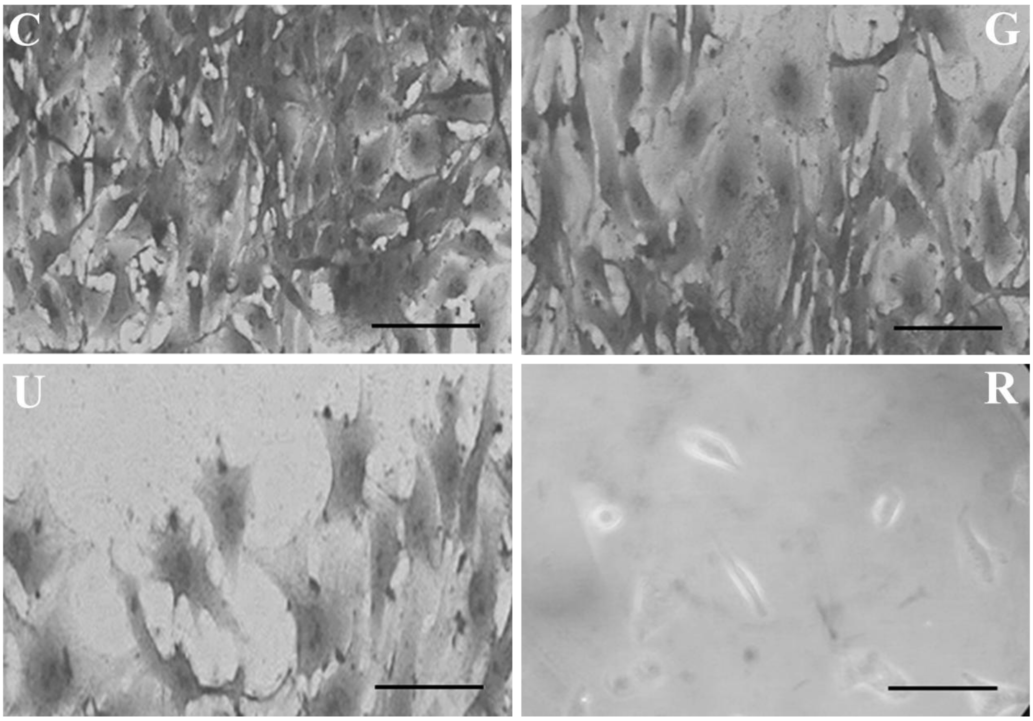

Fig. S4. Phase-contrast microscopic studies of MDA-MB-231 breast cancer cells upon treatment with neem seed extracts. Morphological changes of MDA-MB-231 breast cancer cells were recorded as treated with control drug gemcitabine (G), unripe (U) and ripe (R) neem seed extracts for 24 h at their IC_{50} doses. Cancer cells without addition of drug or seed extracts were considered as posititive control (C). Cells were cultured upto 70-80% confluence before addition of the drug and/or neem seed extracts. Scale bar: 50 µm.
